# Nisin Mutant Prevention Concentration and the Role of Subinhibitory Concentrations on Resistance Development by Diabetic Foot *Staphylococci*

**DOI:** 10.3390/antibiotics11070972

**Published:** 2022-07-19

**Authors:** Margarida Costa, Cláudia Meirinhos, Eva Cunha, Diana Gomes, Marcelo Pereira, Ricardo Dias, Luís Tavares, Manuela Oliveira

**Affiliations:** 1 CIISA—Centro de Investigação Interdisciplinar em Sanidade Animal, Faculdade de Medicina Veterinária, Universidade de Lisboa, Av. da Universidade Técnica de Lisboa, 1300-477 Lisboa, Portugal; margaridaxavicosta@hotmail.com (M.C.); claudianunes97@live.com.pt (C.M.); dgomes@fmv.ulisboa.pt (D.G.); ltavares@fmv.ulisboa.pt (L.T.); moliveira@fmv.ulisboa.pt (M.O.); 2 Laboratório Associado para Ciência Animal e Veterinária (AL4AnimalS), 1300-477 Lisbon, Portugal; 3 Biosystems and Integrative Sciences Institute, Faculdade de Ciências, Universidade de Lisboa, Campo Grande, 1749-016 Lisboa, Portugal; mlpereira@fc.ul.pt (M.P.); rpdias@fc.ul.pt (R.D.)

**Keywords:** diabetic foot infection, nisin, mutant selection window, horizontal gene transfer, MEGA-plate, whole-genome sequencing

## Abstract

The most prevalent microorganism in diabetic foot infections (DFI) is *Staphylococcus aureus*, an important multidrug-resistant pathogen. The antimicrobial peptide nisin is a promising compound for DFI treatment, being effective against *S. aureus*. However, to avoid the selection of resistant mutants, correct drug therapeutic doses must be established, being also important to understand if nisin subinhibitory concentrations (subMIC) can potentiate resistant genes transfer between clinical isolates or mutations in genes associated with nisin resistance. The mutant selection window (MSW) of nisin was determined for 23 DFI *S. aureus* isolates; a protocol aiming to prompt *vanA* horizontal transfer between enterococci to clinical *S. aureus* was performed; and nisin subMIC effect on resistance evolution was assessed through whole-genome sequencing (WGS) applied to isolates subjected to a MEGA-plate assay. MSW ranged from 5–360 μg/mL for two isolates, from 5–540 μg/mL for three isolates, and from 5–720 μg/mL for one isolate. In the presence of nisin subMIC values, no transconjugants were obtained, indicating that nisin does not seem to promote *vanA* transfer. Finally, WGS analysis showed that incubation in the presence of nisin subMIC did not promote the occurrence of significant mutations in genes related to nisin resistance, supporting nisin application to DFI treatment.

## 1. Introduction

Diabetic foot infections (DFI) are one of the major complications of *Diabetes mellitus* [1,2]. DFI are characterized by their polymicrobial feature, being *Staphylococcus aureus* the most frequently isolated species [3,4]

One of the biggest concerns about the treatment of *S. aureus* infections is the resistance ability of this bacterial species to antimicrobials action. Since Methicillin Resistant *Staphylococcus aureus* (MRSA) strains are also usually resistant to other classes of β-lactam antibiotics, vancomycin became one of the alternatives available for the treatment of infections caused by these strains [5,6]. However, *S. aureus* soon became resistant to vancomycin, through conjugation with enterococci followed by transfer of *vanA*, which can be located in the chromosome or in a plasmid. Researchers believe that vancomycin-resistant *S. aureus* (VRSA) develop due to single and independent acquisitions of *Enterococcus* Tn1546 transposon, which carries *vanA*, by MRSA from clonal complex 5 (CC5). Almost all VRSA isolated so far were obtained from patients with DFI [7,8,9]. In 2013, Zhu et al. associated the transfer of the transposon Tn1546, which contains the *vanA* operon, from *Enterococcus* to *S. aureus*, with the pSK41-like plasmid, a class of conjugative staphylococci plasmids that can integrate multiple mobile genetic elements [10,11].

The development of new DFI treatments is mandatory, representing a challenge to the scientific community [12]. Antimicrobial peptides (AMP) represent a promising strategy, being oligopeptides naturally produced by prokaryotes and eukaryotes as part of their innate immune response against several microorganisms [13,14,15].

Nisin, produced by *Lactococcus lactis* subsp. *lactis*, is one of the better described AMP [16,17,18]. Nisin is an amphiphilic peptide with five lanthionine rings and a positive overall charge [16,17]. It acts by two independent mechanisms, producing pores on the bacterial membrane and blocking cell wall synthesis [17,19,20]. Inhibition of cell wall synthesis and pore formation is promoted through the bonding of nisin with lipid II, a peptidoglycan subunit, although nisin may disturb the membrane independently of the lipid II presence [19,21]. 

Nisin has been used in the food industry for 90 years, being a promising product for biomedical applications [22]. It was also demonstrated to be effective against a wide range of Gram-positive bacteria, including antimicrobial resistant strains, such as MRSA, vancomycin-resistant enterococci (VRE), and VRSA [19,20,21]. Bacterial biofilms are also susceptible to this AMP, pointing out its potential use against biofilm-related infections, such as DFI [23,24]. 

As previously referred, antimicrobial resistance has been a growing problem in the last decades as demonstrated by the increment in reported resistances. To avoid the administration of doses that could promote a selective mutant environment, Zhao and Drlica proposed the mutant selection window (MSW) concept [25], referring to an antibiotic concentration range that has as a lower limit the minimum inhibitory concentration (MIC) and as the higher limit the mutant prevention concentration (MPC) [26]. The MIC is the lowest concentration of an antimicrobial that inhibits the growth of the majority of the susceptible cells, while the MPC is the concentration that inhibits the growth of the least susceptible mutant [27]. These are usually single-step mutants, since for a cell to multiply in the presence of antibiotic concentrations above MPC values would require the simultaneous occurrence of two or more mutations, which is a rare event [28].

The use of AMP for human infections’ treatment can trigger different responses by different bacteria [29]. In the presence of high concentrations of AMP, cell membranes can be damaged and break down, but at lower concentrations, peptides can translocate to the cytoplasm and have interactions with the ribosome and with the DNA at an electrostatic level [30,31]. In fact, when used in non-lethal subMIC concentrations, AMP can have an impact on the expression of the bacterial genome and affect bacteria virulence and resistance [29].

As such, the main goals of this work were to determine the MSW of nisin regarding a collection of *S. aureus* clinical isolates obtained from patients with DFI; to evaluate if subinhibitory concentrations of nisin and vancomycin could prompt the horizontal transfer of *vanA* from *E. faecium* to the *S. aureus* DFI isolates; and to assess the impact of nisin subMIC on DFI isolates’ genome, more specifically on genes related to nisin resistance.

## 2. Results and Discussion

### 2.1. Mutant Selection Window

Nowadays, antibiotic concentrations established in the therapeutic protocols for in vivo administration have the MIC determination as a reference. However, the clinical application of antimicrobial doses based on MIC values could exert a selective pressure on bacteria, allowing the selection of resistant mutants [28]. Although there are few reports of bacterial resistance to nisin, the appearance of some cases reveals the importance of determining the MSW of this AMP. To our knowledge, the determination of the MPC of nisin regarding *S. aureus* DFI isolates has not been previously performed. 

In this study, MSW and MPC determination were performed for all the 23 *S. aureus* isolates and for the reference strain *S. aureus* ATCC 29213, using 10^10^ CFU/mL bacterial suspensions. Determinations were performed twice, as previously reported [32,33,34]. This concentration was selected based on the fact that the usual bacterial concentration found in infections, around 10^5^ CFU/mL per gram of tissue, is five-fold lower than the concentration used in this protocol, which guarantees that the antimicrobial concentration obtained will be able to eliminate all the bacteria present in in vivo infections [8]. The mean MPC values obtained ranged from 360 µg/mL to more than 720 µg/mL (Table 1). Nisin MPC mean values were 360 µg/mL for 8.33% of the tested isolates (n = 2), 540 µg/mL for 12.5% of the isolates (n = 3) and 720 µg/mL for 4.17% (n = 1) of the isolates. MPC value could not be determined regarding 18 isolates (75%), since they were able to grow in the presence of the highest concentration of nisin tested (720 µg/mL). Considering the minimum inhibitory concentration (MIC) values previously described for these isolates [35], the MSW ranged from 5 to 360 μg/mL for two isolates, from 5 to 540 μg/mL for three isolates, and from 5 to 720 μg/mL for one isolate. The MPC/MIC ratios varied from 29 to 144 (Table 1).

The MPC values and MPC/MIC ratio obtained in this study are in accordance with a previously reported study which determined that the vancomycin MPC_80_ value for 855 *S. aureus* clinical isolates was 64 times higher than the MIC_80_ value [36]. Both compounds, vancomycin and nisin, act at the same target, the lipid II, although with a different mode of action [37]. Vancomycin inhibits the cell wall synthesis by binding to the sequence of the C-terminal D-ala-D-ala of the lipid II, while on the other hand, the lanthionine rings of nisin bind to the pyrophosphate of lipid II, using it as a docking molecule to form pores on the target membranes [37,38]. In fact, other authors already observed a high MPC variation between bacteria and drugs, and suggested that, considering the high variability of MPC values for a given bacterial strain–antimicrobial combination, MPC values should be understood as a range with confidence intervals, rather than an absolute value [39].

Despite these results regarding the MSW determination, the potential of nisin as a biomedical repurposed agent remains a reality. In a study performed in 2008, nisin was applied to the nipple and mammary areola of four women with clinical signs of mastitis by *S. aureus*, at concentrations selected based on the 2006 European Food Safety Authority (EFSA) report on nisin toxicity after oral administration, which referred that the acceptable daily intake (ADI) of nisin was 0.13 mg/kg body weight [40]. Since the nipples presented infected fissures (infected wound) and no signs of toxicity were observed after nisin application, the more recent nisin ADI established by EFSA was also considered in our study for comparison purposes. The updated nisin ADI value is 1 mg/kg body weight [41], which means that a person with medium weight (65 kg) can ingest a maximum of 65 mg of nisin per day. Considering that in this study nisin MPC values regarding most isolates were higher than 720 µg/mL, if 2 mL of a biogel supplemented with nisin at this concentration was applied to a DFI 3 times a day, this would correspond to the application of 4 mg of nisin to the wound, which is 16 times below the ADI for a medium weight individual.

### 2.2. Horizontal Gene Transfer

Horizontal gene transfer (HGT) is related to the emergence and dissemination of highly resistant strains, such as *S. aureus* and enterococci, being these microorganisms classified by the WHO as high-priority pathogens due to their resistance ability [42]. One example is the emergence of VRSA since vancomycin is often a last resort antibiotic applied in the treatment of several infections promoted by resistant bacteria, including DFI [43,44,45].

In our study, the HGT of *vanA* to *S. aureus* from our DFI collection was evaluated, in the absence of environmental pressure and in the presence of subinhibitory concentrations of nisin and vancomycin (Table 2). Initially, a multiplex PCR confirmed the absence of the *vanA* gene in all the *S. aureus* isolates under study, allowing to use the 23 clinical isolates as recipients to evaluate the occurrence of HGT.

**Figure 1:**
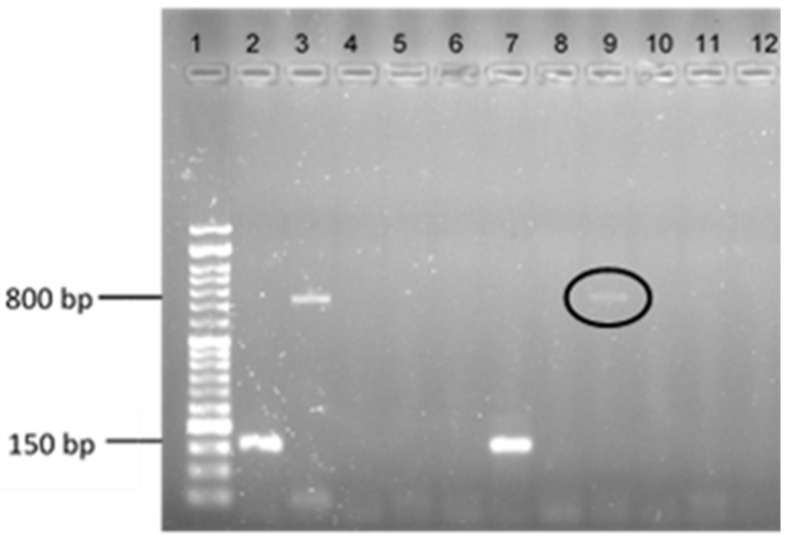
Electrophoresis results of a multiplex PCR reaction to determine the presence of the *vanA* gene in the transconjugants obtained after MPC protocol. Lane 1: ladder VI (Nzytech®, Lisbon, Portugal). Lane 2: *mecA* positive control *S. aureus* 01-00694; Lane 3: *vanA* positive control *Enterococcus faecium* CCUG 36804; Lane 4: negative control; Lane 5: *S. aureus* ATCC 29213; Lanes 6 to 12: the *S. aureus* DFI clinical isolates under study in the following order: A6.3, B14.2, Z2.2, Z5.2, Z17.2, Z.27.2, Z32.2.

Since the pSK41 plasmid has been described as required for the transfer of the *vanA* gene from enterococci to staphylococci, a PCR analysis was performed regarding all the clinical *S. aureus* isolates to evaluate the presence of this plasmid. Results showed that none of the isolates under study presented the pSK41 plasmid, in spite of it being already detected in several staphylococci strains, including in isolates responsible for community-acquired MRSA (CA-MRSA) (e.g., CC8) and hospital-acquired MRSA infections (HA-MRSA) (e.g., CC5). Therefore, the HGT protocol was performed using the 23 *S. aureus* clinical isolates and the reference strain as potential recipients, and the *E. faecium* CCUG 36804 as the *vanA* gene donor. After the mating experiments, PCR analysis was performed regarding all the possible transconjugants recovered. A band matching the *vanA* positive control was obtained from one transconjugant (4.17%, n = 1/24) resulting from the mating between the recipient *S. aureus* Z5.2 and the donor *E. faecium* CCUG 36804 (Figure 1 and Table 2) in the absence of environmental pressure. Then, the obtained PCR product was evaluated by DNA sequencing, allowing to confirm the presence of the *vanA* gene in the transconjugant.

The receptor DFI isolate *S. aureus* Z5.2 is a methicillin-susceptible *S. aureus* (MSSA) and belongs to clonal complex 5, as the majority of the clinical isolates under study (69.5%) (Table 1) [46]. Clones belonging to the CC5 are the predominant cause of HA-MRSA, being also present in the community. Additionally, the majority of the VRSA strains reported so far belong to CC5 [47,48]. Another interesting fact is the methicillin-susceptible profile of the transconjugant *S. aureus* Z5.2, since almost all the VRSA reported are also MRSA [49,50]. The association between the emergence of VRSA and MRSA is probably associated with vancomycin treatment being only recommended when semi-synthetic penicillin fail, which indicates the presence of methicillin-resistant mutants at the site of infection when the new vancomycin-based antibiotherapy is started. Results from this study seem to indicate that the MSSA strains also have the ability to acquire other resistant determinants besides *mecA*.

The fact that CC5 strains are repeatedly acquiring resistance to vancomycin is probably related to some predisposition of these strains to HGT [8]. In our study, the acquisition of *vanA* was accomplished by a CC5 MSSA not presenting pSK41, which supports the hypothesis that other plasmids could be related to the transfer of the *vanA* gene.

In addition, two matting experiments were performed using the same isolates, in the environmental pressure conditions caused by the presence of nisin and vancomycin, both at subMIC concentrations. None of these experiments allowed the recovery of transconjugants. Similar results were obtained by Cunha et al. [39], who used subMIC concentrations of nisin, as environmental pressure, and no *vanA* transfer was observed. These were interesting results, since it has been described that the presence of subMIC values of antibiotics promotes HGT and the emergence of resistant bacteria [51]. However, *vanA* HGT is a complex process, not entirely clarified, that may also be facilitated by some molecules, such as pheromone-inducible surface proteins and specific plasmids [39]. Further studies on this issue will help to understand this process.

### 2.3. MEGA-Plate Assays and Variant Call Analysis

To evaluate the influence of nisin subinhibitory concentrations on the *S. aureus* genome, short-read genome sequencing of the isolates collected during the MEGA-plate assays was performed at BioISI Genomics for variant call analysis (Supplementary file). A total of 21 isolates were obtained in the MEGA-plate assays, from which 7 isolates were collected in the first assay, 8 isolates in the second assay, and 6 isolates in the third assay (Table 3).

**Figure 2:**
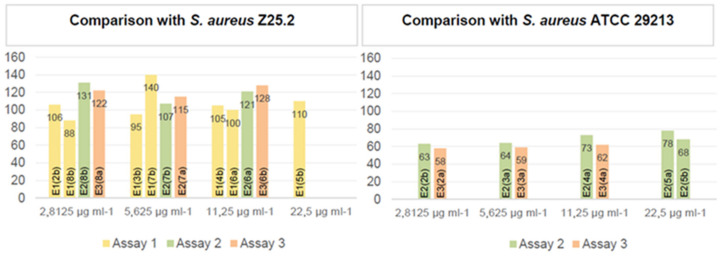
Graphical representation of the number of genes from each sequenced isolate that presented mutations, per assay and per MEGA-plate division from which they were collected, when compared to the original strain from which they derived. On the left, it is possible to compare each isolate with the original *S. aureus* Z25.2 and, on the right, with the original *S. aureus* ATCC 29213. E1—assay 1; E2—assay 2; E3—assay 3.

Comparing the sequences of the original isolates and the ones obtained throughout the MEGA-plate assays, it was possible to assess the number of genes that presented mutations (Figure 2). 

**Figure 3:**
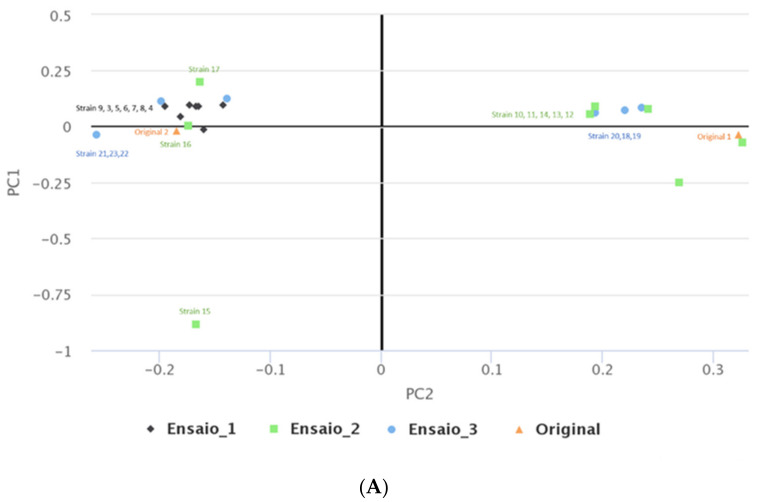

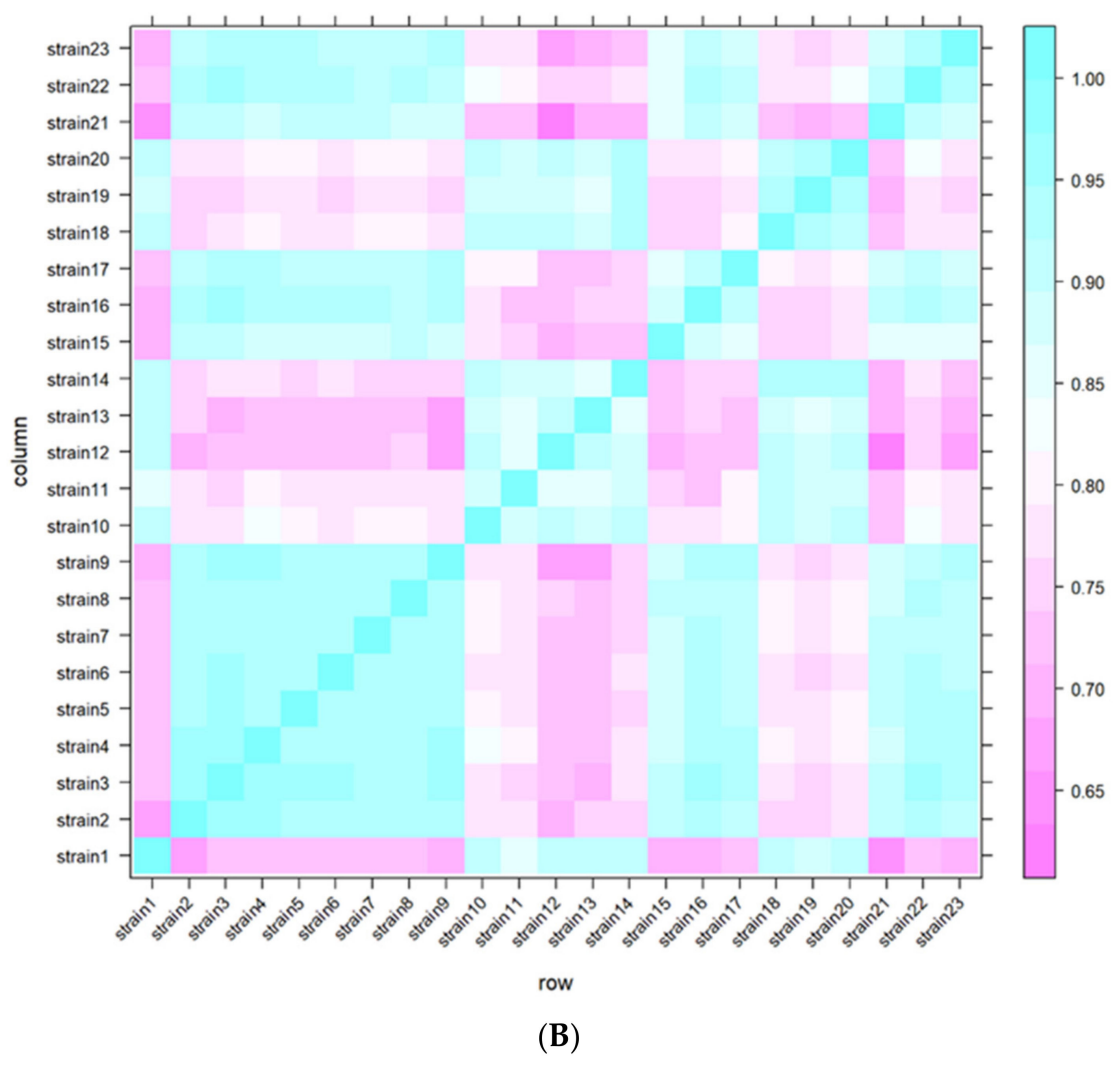
Different plots representing the differentiation and/or similarity between the *S. aureus* isolates collected and sequenced in this study, including the original strains (*S. aureus* ATCC 29213 and *S. aureus* Z25.2). (**A**)—multidimensional scaling (MDS) plot of the *S. aureus* isolates sequenced, including of the original strains; (**B**)—heatmap representing the similarity of the different isolates. The intensity of the blue color represents the level of similarity between isolates, while the intensity of the pink color represents the level of dissimilarities between isolates.

Figure 3 shows the number of genes that presented mutations detected after comparison with the original *S. aureus* Z25.2 (original 2, left graphic) and *S. aureus* ATCC 29213 (original 1, right graphic) strains, grouped according to the MEGA-plate division from which they were collected.

It was possible to observe that isolates derived from the original *S. aureus* ATCC 29213 presented far less genetic mutations (with the number of mutated genes ranging between 58 and 78), than the ones derived from the original *S. aureus* Z25.2 DFI isolate (with the number of mutated genes ranging from 88 to 140). It was not possible to detect a pattern regarding the number of mutated genes and the different assays or the nisin concentration to which they were exposed. However, in the isolates derived from *S. aureus* ATCC 29213, the number of genes that presented mutations tended to increase in isolates collected from divisions closer to the middle MEGA-plate division, where nisin’s concentration was the highest used in this study (MIC).

The analysis performed in this study focused on the evaluation of specific mutations in genes involved in *S. aureus* resistance to nisin. Currently, the glycopeptide resistance-associated two-component system (GraSR) has been identified as a very important two-component system in *S. aureus* known for being involved in nisin resistance [52,53]. GraSR is mainly responsible for controlling the *vraFG* operon, which is positioned right downstream of the *graXRS* genes, ultimately encoding for an ABC transporter that plays a huge role regarding resistance to cationic AMP [53]. One of the genes of this system encodes for the GraX protein, an essential component of this system. This two-component system was first discovered when investigators saw that its overexpression led to a higher vancomycin resistance; but to date, it has already been associated with the expression of the *mprF* gene and other operons [52]. Additionally, this system was shown to have an impact on biofilm formation [54]. In this study, only one isolate presented a mutation in a gene of this complex, *graR*, namely an isolate derived from the *S. aureus* Z25.2 DFI isolate, which was collected in the first assay from a division where nisin’s concentration was at its lowest (2.8125 μg mL^−1^). Moreover, El Shazely et al. [55], who studied the resistance evolution in *S. aureus,* observed mutations in the *vraF* gene in strains submitted to subMIC concentrations of the AMP pexiganan, and also in other genes associated with detoxification by efflux, which may be associated with nisin resistance. 

Another two-component system of great importance regarding bacitracin and nisin resistance is NsaRS, also known as BraRS [53,56,57]. Nisin resistance often comes associated with mutations in the *nsaS* gene, which encodes for a sensor kinase. A study conducted by Arii et al. showed that the exposure of *S. aureus* to sub-inhibitory concentrations of nisin led to the occurrence of spontaneous mutations in genes associated with this system, which resulted in higher resistance to nisin [58], through mechanisms yet unknown [56]. In this study, no mutations were detected in any genes related to this system, allowing to conclude that isolates’ exposure to nisin subMIC values did not promote genomic alteration that conferred resistance to nisin.

In conclusion, results showed that, to avoid the development of resistant mutants, nisin therapeutic doses should be established based on the MSW, that nisin does not seem to promote *vanA* transfer, and that nisin subMIC values do not prompt significant mutations in genes related to nisin resistance. As such, nisin seems to be a good candidate to be applied to DFI treatment in the future.

## 3. Materials and Methods

### 3.1. Bacterial Isolates

In this study, a collection of 23 *S. aureus* DFI isolates was used. These isolates were previously collected from patients with DFI [7] and further selected and characterized in terms of clonality, antimicrobial resistance, and virulence profiles [46]. Additionally, the reference strain *S. aureus* ATCC 29213 was also included as a control. Each isolate was maintained at −80 °C in buffered peptone water with 20% of glycerol throughout the duration of this study.

### 3.2. Nisin Solution

A nisin (Sigma-Aldrich, St. Louis, MO, USA) with a purity of 2.5% (1000 IU/mg) was used. To obtain a 1000 µg/mL stock solution, 1 g was dissolved in 25 mL of 0.02M HCl (Merck, Darmstadt, Germany) [35]. Afterwards, nisin was sterilized by filtration using a 0.22 μm filter (Frilabo, Maia, Portugal) and stored at 4 °C. 

### 3.3. Determination of the Mutant Prevention Concentration 

A modified version of the protocol described by Sinel et al. [59] was used to determine the MPC of nisin regarding the 23 *S. aureus* DFI isolates under study.

Each isolate was inoculated in brain heart infusion (BHI) agar (VWR Chemicals, Leuven, Belgium), and, after a 24 h incubation at 37 °C, a suspension of 0.5 MacFarland (1 × 10^8^ CFU/mL) was performed and used to inoculate two BHI agar plates. After a 24 h incubation at 37 °C, the bacterial lawn was collected from the two BHI plates and resuspended in 1mL of BHI broth to achieve a bacterial suspension with a concentration of 10^10^ CFU/mL. To confirm the concentration values of the bacterial suspensions, these were serially diluted for viable cell count. Afterwards, 50 µL of the original 10^10^ CFU/mL suspensions was inoculated in Mueller–Hinton agar (MHA) (Oxoid, Hampshire, UK) supplemented with the following concentrations of nisin: 5.63, 11.25, 22.5, 45, 90, 180, 360, and 720 µg/mL. These concentrations were selected considering a two-fold increase in the mean MIC value (11.25 µg/mL) that was previously determined [35]. A subMIC value was also included (5.63 µg/mL). Finally, plates were incubated for 72 h at 37 °C for MPC determination.

The MPC corresponded to the minimum concentration of nisin that prevented the growth of resistant mutants after the incubation period. For each isolate, the mutants grown in the presence of nisin concentrations below the MPC were isolated and stored at −80 °C in a solution of buffered peptone water with 20% glycerol (VWR Chemicals, Leuven, Belgium). The MPC values of nisin were determined in two independent rounds.

### 3.4. Horizontal Gene Transfer

#### 3.4.1. DNA Extraction

DNA extraction was performed according to the protocol described by Mottola et al. [46]. All isolates were inoculated in BHI agar and incubated for 24 h at 37 °C. Then, four to five bacterial colonies were collected using a sterile loop and resuspended in 100 μL of TBE buffer (0.9 M Tris-Borate, 0.01 M EDTA, pH 8.3, Omega, Norcross, GA, USA) supplemented with 0.1% Tween 20 (Merck, Darmstadt, Germany) solution. After homogenization, the suspension was incubated for seven minutes at 97 °C, centrifuged at 15,000 rpm for 5 min, and the supernatant was collected for PCR screening.

#### 3.4.2. Multiplex PCR for vanA Detection

Before the horizontal gene transfer protocol, it was necessary to confirm the absence of *vanA* gene in the collection of 23 *S. aureus* DFI isolates under study, using multiplex PCR [60]. Two pairs of primers, synthesized by STABVIDA® (Lisbon, Portugal), targeting *vanA* (5′ GGG AAA ACG ACA ATT GC 3′) and *mecA* (5′ TCCAGAT-TACAACTTCACCAGG 3′) were used [46,60].

The PCR mixture had a final volume of 28.5 μL, including 10 μL of the Supreme NZYTaq 2x Green Master Mix (NZYTech, Lisbon, Portugal), 0.29 μL of the *vanA* primer (0.5 µM), 0.23 μL of the *mecA* primer (0.4 µM), 12.46 μL of PCR-grade water and 5 μL DNA template (170 ng/μL).

PCR amplification was completed in a MyCycler Thermal Cycler (BioRad, Lisbon, Portugal) using the following conditions: initial denaturation at 94 °C for 4 min; 10 cycles including denaturation at 94 °C for 30 s, annealing at 64 °C for 30 s, and elongation at 72 °C for 45 s; 25 cycles comprising denaturation at 94 °C for 30 s, annealing at 50 °C for 45 s and elongation at 72 °C for 2 min, and a final extension step at 72 °C for 10 min.

An electrophoresis gel was performed to perceive the amplified products, using a 1.5% agarose gel (NZYTech, Lisbon, Portugal) and a buffer stained with GreenSafe (NZYTech, Lisbon, Portugal) at 90 V for 45 min. A molecular weight marker, NZYDNA ladder VI (NZYTech, Lisbon, Portugal), was also included. Results were visualized by transillumination (ChemiDoc XRS+, BioRad, Lisbon, Portugal).

Two positive control strains, *Staphylococcus aureus* 01-00694 (*mecA* positive) and *Enterococcus faecium* CCUG 36804 (*vanA* positive), were included in each PCR amplification protocol, as well as a negative control, with no DNA.

#### 3.4.3. Horizontal Gene Transfer protocol

**Figure 4:**
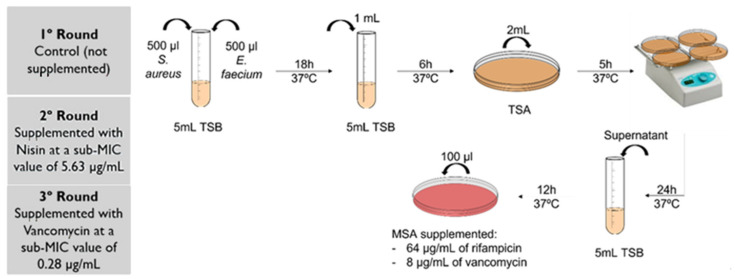
Schematic representation of the adapted horizontal gene transfer protocol. TSB—tryptic soy broth; TSA—tryptic soy agar; MSA—mannitol salt agar.

To test if nisin selective pressure induced horizontal gene transfer, a protocol adapted from Niederhäusern et al. [61] was performed (Figure 4). Mating experiments were performed in three rounds, using the VRE rifampicin-susceptible (Van^R^ Rif^S^) *E. faecium* CCUG 36804 strain as a donor of the *vanA* gene [39], and as recipients of all the 23 *S. aureus* DFI isolates of our collection. Initially, resistance to rifampicin was induced in the recipients, as described elsewhere [61], allowing to obtain strains resistant to rifampicin and susceptible to vancomycin (Van^S^ Rif^R^)

After performing a 0.5 MacFarland suspension for each isolate, 500 μL of the donor and 500 μL of each one of the recipients were added to 5 mL of non-supplemented TSB (tryptic soy broth, VWR Chemicals, Leuven, Belgium) and incubated at 35 °C for 18 h. After incubation, 1 mL of the bacterial suspension was added to 5 mL of TSB and further incubated for 6 h at 37 °C. Afterwards, 2 mL of each suspension was inoculated in TSA (tryptic soy agar, VWR Chemicals, Leuven, Belgium) and incubated for 5 h at 37 °C on a shaker, to promote mating. Then, the plates were incubated at 37 °C for 24 h, after which the bacterial suspension that remained at the surface of the agar plates was removed and inoculated in 5 mL of TSB. After an incubation period of 12 h at 37 °C, 100 μL of the suspension was inoculated in mannitol salt agar (MSA, PanReac AppliChem, Barcelona, Spain) supplemented with 64 μg/mL of rifampicin and 8 μg/mL of vancomycin (vancomycin hydrochloride, Abcam, Cambridge, UK) to allow Van^R^Rif^R^ transconjugants selection. Transconjugants were stored at −80°C in buffered peptone water with 20% glycerol and a PCR analysis was performed to confirm the presence of the *vanA* gene in these isolates. Moreover, strains with a positive *vanA* PCR were submitted for DNA Sanger sequencing by STABVIDA® (Lisbon, Portugal), to confirm *vanA* gene transference.

This first mating round was performed with non-supplemented TSB, as control. Then, a second mating round was performed in the presence of nisin, with all media used being supplemented with nisin at subMIC (5.63 μg/mL). A third mating round was also performed as a control, in the presence of a subMIC value of vancomycin (0.28 μg/mL), selected based on the previous MIC determination [46].

#### 3.4.4. PCR for pSK41-like Plasmid Detection

To evaluate the presence of the pSK41-like plasmid in the 23 clinical isolates under study, a PCR protocol was performed, using a pair of primers targeting *traE* (5′ ACA AAT GCG TAC TAC AGA CCC TAA ACG A 3′), synthesized by STABVIDA® (Lisbon, Portugal) [11,62].

The PCR mixture had a final volume of 50 μL, including 10 μL of the Supreme NZYTaq 2x Green Master Mix (NZYTech, Lisbon, Portugal), 0.4 μL of *traE* primer (0.4 uM), 34.2 μL of PCR-grade water, and 5 μL DNA template (170 ng/μL). PCR amplification was completed in a MyCycler Thermal Cycler (BioRad, Lisbon, Portugal) using the following conditions: initial denaturation at 94 °C for 2 min; 30 cycles comprising denaturation at 95 °C for 15 s, annealing at 53 °C for 90 s and elongation at 72 °C for 90 s, and a final extension step at 72 °C for 7 min. An electrophoresis gel was performed to perceive the amplified products, using a 1.5% agarose gel (NZYTech, Lisbon, Portugal) and a buffer stained with GreenSafe (NZYTech, Lisbon, Portugal) at 90 V for 45 min. A molecular weight marker, NZYDNA ladder VII (NZYTech, Lisbon, Portugal), was also included. Results were visualized by transillumination (ChemiDoc XRS+, BioRad, Lisbon, Portugal).

A positive control strain, *Staphylococcus aureus* RN4220 (pGO1 positive), gently provided by Dr. Alex O’Neill from the University of Leeds, was included in the PCR amplification protocol, as well as a negative control, with no DNA [63].

### 3.5. MEGA-Plate Assays and Variant Call Analysis

**Figure 5:**
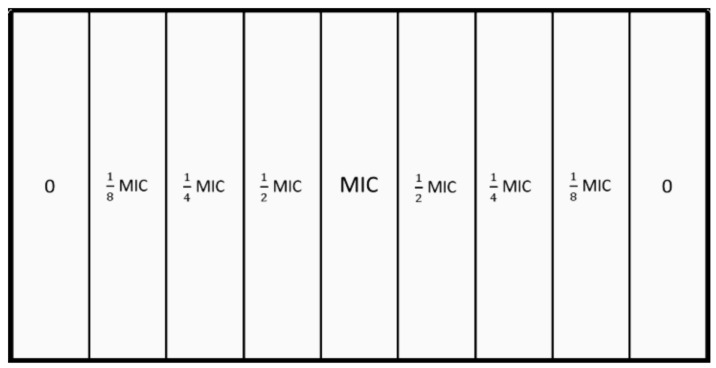
Schematic representation of the different nisin concentrations tested in each internal division of the MEGA plate. The MIC value of 22.5 μg/mL was previously assessed. The ½ MIC division corresponds to a nisin concentration of 11.25 μg/mL, the ¼ MIC division corresponds to a nisin concentration of 5.625 μg/mL, and the 1/8 MIC division corresponds to a nisin concentration of 2.8125 μg/mL. Suspensions were inoculated along the plate extremity, at division 0.

Three assays were performed using the microbial evolution and growth arena (MEGA)-plate described by Baym et al. [64], using a glass plate with 90 × 45 × 5 cm and 9 equal internal divisions of 45 × 10 × 3 cm and a glass cover (Figure 5). Prior to its use, the MEGA-plate was sterilized with ethanol (96%) and exposed to UV light for 2 h in a laminar flow chamber. The divisions were later filled with nonselective brain heart infusion (BHI) medium (VWR® Chemicals, Belgium), supplemented with bacteriological agar (VWR® Chemicals, Belgium) at 1.5 % (15 g/L) and with different nisin concentrations (2.8125 µg/mL, 5.625 µg/mL, 11.25 µg/mL, and 22.5 µg/mL, corresponding to nisin 1/8 MIC, ¼ MIC, ½ MIC, and MIC values (Figure 5). Additionally, semi-solid BHI media (BHI broth with bacteriological agar at 8 g/L), supplemented with amphotericin B (2 µg/mL), was prepared and poured on top of the previous medium.

**Figure 6:**
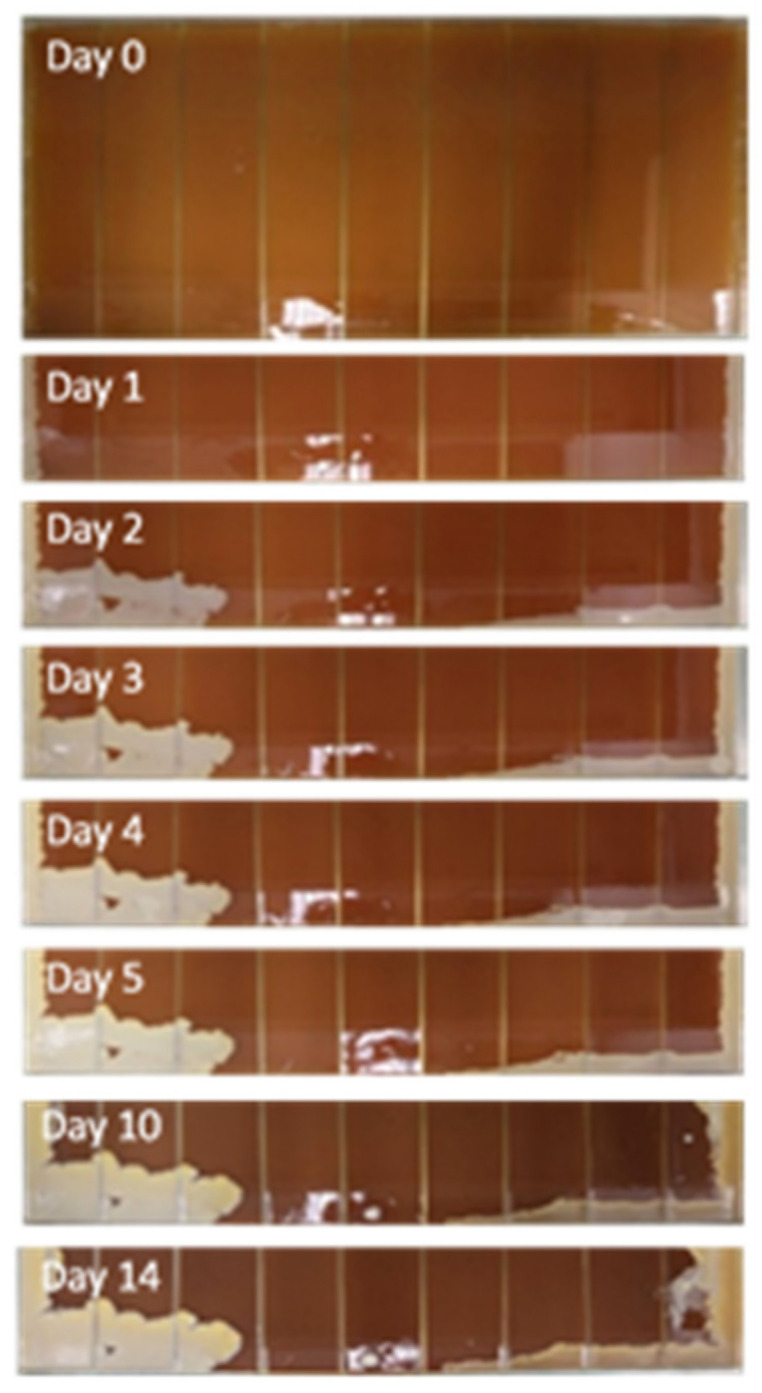
Photographic record of one of the MEGA-plate’s assays, in which it is possible to assess the multiplication through time and space of *S. aureus* ATCC 29213 on the left side of the plate and of *S. aureus* Z25.2 on the right side of the plate.

Then, a suspension of the reference strain *S. aureus* ATCC 29213 (with 10^8^ CFU/mL) was inoculated on the left extremity of the MEGA-plate, and a suspension of a selected DFI isolate, *S. aureus* Z.25.2, was inoculated on the right extremity, in an area of 1 cm of thickness. The plates were incubated at 37 °C for a period of 15 days and, each day, the growth of the staphylococci was accessed and registered via time-lapse photography twice a day using a Canon® M50 camera, in order to evaluate the bacterial multiplication progress through time (Figure 6).

After incubation, isolates were collected from each division and inoculated in BHI agar medium plates. After a 24 h incubation at 37 °C, isolates’ identification was confirmed through Gram staining, catalase and coagulase tests, and inoculation on MSA. This protocol was performed in three independent assays.

All Isolates obtained were analyzed by whole-genome sequencing using Illumina MiSeq paired-end short read sequencing at Biosystems and Integrative Sciences Institute (BioISI) Genomics. It was obtained between 0.6 and 1.3 million reads, corresponding to a sequencing depth between 34 and 105 times the length of the *S. aureus* genome, respectively. The reads length ranged between 157 bps and 227 bps, and the quality of the sequenced reads ranged between a Phred score of 30.63 and 38.06. A customized analytical pipeline developed by BioISIGenomics® for short-read genomic sequencing was used. During the pre-processing stage, the quality of the reads was assessed using FASTQC v0.11.9 [65]. Trimmomatic v0.38 was used to trim and filter the reads according to their length and quality score [66]. The alignment against the ENSEMBL reference sequences: *Staphylococcus aureus* Newman strain (genome assembly: ASM1046v1); and *S. aureus* ATCC 29213 (genome assembly: ASM126771v2), was performed using Bowtie2 v.2.4.1 [67]. The mapping was performed against the *S. aureus* Newman strain because, contrary to the Z25.2 strain, the Newman strain is fully sequenced and annotated. Variant calling was performed using Mpileup (Samtools v 1.10) [68]. The variant call events were filtered according to the sequencing depth (DP > 100) and to an estimate of the probability of observing a call plainly by chance (%QUAL ≥ 20). The original strains inoculated on the MEGA-plate (*S. aureus* Z.25.2 and *S. aureus* ATCC 29213) and the isolates collected in the assays were compared using VCFTools v0.1.16 [69]. ENSEMBL Variant Effect Predictor tool (release/100.2) was used to annotate the VCF files obtained in the previous step [70], within QuickGO release-1.0.1 to retrieve the GO terms annotation [71]. Finally, SNiPlay v1.0_TTS toolkit and PLINK v2.3 were used to generate heatmaps (IBS matrix) and multi-dimensional scaling (MDS) plots [72,73].

## Figures and Tables

**Table 1:** Nisin mutant prevention concentration (MPC) values and MPC/MIC ratio for the 24 *S. aureus* isolates under study.

Isolate Identification	*mecA* Gene	Clonal Complex	MPC Value	MPC/MIC Ratio
*S. aureus* A1.1	+	5	>720	-
*S. aureus* A5.2	-	8	>720	-
*S. aureus* A6.3	-	7	>720	-
*S. aureus* B3.2	-	5	>720	-
*S. aureus* B3.3	-	5	>720	-
*S. aureus* B7.3	+	5	>720	-
*S. aureus* B13.1	+	5	720	144
*S. aureus* B14.2	+	22	>720	-
*S. aureus* Z1.1	+	22	>720	-
*S. aureus* Z2.2	-	5	>720	-
*S. aureus* Z3.1	-	5	>720	-
*S. aureus* Z5.2	-	5	>720	-
*S. aureus* Z12.2	-	5	540	43
*S. aureus* Z14.1	-	5	>720	-
*S. aureus* Z16.1	+	5	>720	-
*S. aureus* Z17.2	-	30	360	72
*S. aureus* Z21.1	+	5	>720	-
*S. aureus* Z21.3	+	5	>720	-
*S. aureus* Z23.2	-	45	540	108
*S. aureus* Z25.2	-	182	540	43
*S. aureus* Z27.2	-	5	>720	-
*S. aureus* Z27.3	-	5	>720	-
*S. aureus* Z32.2	-	5	360	29
*S. aureus* ATCC 29213	-	?	>720	-

**Table 2:** Results obtained after the protocol of horizontal gene transfer in each matting round performed.

Isolate Identification	Control (Not Supplemented)	2 Round Supplemented with Nisin (Sub-MIC Value of 5.63 µg/mL)	3 Round Supplemented with Vancomycin (Sub-MIC Value of 0.28 µg/mL)
*S. aureus* A1.1	-	-	-
*S. aureus* A5.2	-	-	-
*S. aureus* A6.3	-	-	-
*S. aureus* B3.2	-	-	-
*S. aureus* B3.3	-	-	-
*S. aureus* B7.3	-	-	-
*S. aureus* B13.1	-	-	-
*S. aureus* B14.2	-	-	-
*S. aureus* Z1.1	-	-	-
*S. aureus* Z2.2	-	-	-
*S. aureus* Z3.1	-	-	-
*S. aureus* Z5.2	+ (*vanA* detected)	-	-
*S. aureus* Z12.2	-	-	-
*S. aureus* Z14.1	-	-	-
*S. aureus* Z16.1	-	-	-
*S. aureus* Z17.2	-	-	-
*S. aureus* Z21.1	-	-	-
*S. aureus* Z21.3	-	-	-
*S. aureus* Z23.2	-	-	-
*S. aureus* Z25.2	-	-	-
*S. aureus* Z27.2	-	-	-
*S. aureus* Z27.3	-	-	-
*S. aureus* Z32.2	-	-	-
*S. aureus* ATCC 29213	-	-	-

Legend: - *vanA* gene not detected; + *vanA* gene detected.

**Table 3:** Origin of the *S. aureus* isolates collected in the MEGA-plates assays.

Isolate Code	Assay	Original *S. aureus* Strain	MEGA-Plate Division Concentration
E1(2b)	1	*S. aureus* Z25.2	1/8 MIC-2.8125 µg mL^−1^
E1(3b)	1	*S. aureus* Z25.2	1/4 MIC-5.625 µg mL^−1^
E1(4b)	1	*S. aureus* Z25.2	1/2 MIC-11.25 µg mL^−1^
E1(5b)	1	*S. aureus* Z25.2	MIC-22.5 µg mL^−1^
E1(6a)	1	*S. aureus* Z25.2	1/2 MIC-11.25 µg mL^−1^
E1(7b)	1	*S. aureus* Z25.2	1/4 MIC-5.625 µg mL^−1^
E1(8b)	1	*S. aureus* Z25.2	1/8 MIC-2.8125 µg mL^−1^
E2(2b)	2	*S. aureus* ATCC 29213	1/8 MIC-2.8125 µg mL^−1^
E2(3a)	2	*S. aureus* ATCC 29213	1/4 MIC-5.625 µg mL^−1^
E2(4a)	2	*S. aureus* ATCC 29213	1/2 MIC-11.25 µg mL^−1^
E2(5a)	2	*S. aureus* ATCC 29213	MIC-22.5 µg mL^−1^
E2(5b)	2	*S. aureus* ATCC 29213	MIC-22.5 µg mL^−1^
E2(6a)	2	*S. aureus* Z25.2	1/2 MIC-11.25 µg mL^−1^
E2(7b)	2	*S. aureus* Z25.2	1/4 MIC-5.625 µg mL^−1^
E2(8b)	2	*S. aureus* Z25.2	1/8 MIC-2.8125 µg mL^−1^
E3(2a)	3	*S. aureus* ATCC 29213	1/8 MIC-2.8125 µg mL^−1^
E3(3a)	3	*S. aureus* ATCC 29213	1/4 MIC-5.625 µg mL^−1^
E3(4a)	3	*S. aureus* ATCC 29213	1/2 MIC-11.25 µg mL^−1^
E3(6b)	3	*S. aureus* Z25.2	1/2 MIC-11.25 µg mL^−1^
E3(7b)	3	*S. aureus* Z25.2	1/4 MIC-5.625 µg mL^−1^
E3(8b)	3	*S. aureus* Z25.2	1/8 MIC-2.8125 µg mL^−1^

## Data Availability

The datasets used and/or analyzed during the current study are available from the corresponding author on reasonable request.

## Supplementary Materials

The following supporting information can be downloaded at: https://www.mdpi.com/article/10.3390/antibiotics11070972/s1, Supplementary file.
