# Supplementary material for: Nisin Mutant Prevention Concentration and the Role of Subinhibitory Concentrations on Resistance Development by Diabetic Foot Staphylococci
Source: Antibiotics (Basel). 2022 Jul 19;11(7):972. doi: 10.3390/antibiotics11070972 (PMC9311964; doi:10.3390/antibiotics11070972)
Supplement: Supplementary file 1 [file antibiotics-11-00972-s001.zip › Supplementary_data_legend.pdf]

Supplementary Data S1. Tables containing the annotated variant calling outputs for each pair of samples (1Vs1) using as references ENSEMBL *S. aureus* strain Newman (genome assembly: ASM1046v1) and *S. aureus* strain ATCC 29213 (genome assembly: ASM126771v2). The mutations found in intergenic regions were removed ("upstream_gene_variant" and "downstream_gene_variant"), keeping "gene mutations" events.

Uploaded_variation: organized as "Chromosome"_ "Chromosome start position"_"Allele"; Location: chromosome start/end coordinates in the formats "Chromosome":"start" or "Chromosome":"start"-"end"; Allele: The list of alternative alleles at this position; Gene : Ensembl ID of the affected gene; Feature: Ensembl ID of the feature; Feature_type : annotated feature type. Could be a transcript ("Transcript"), a regulatory feature ("RegulatoryFeature") or a motif ("MotifFeature"); Consequence: consequence type of the mutation (detailed in **Supplementary Table S1**); cDNA_position: relative position of the mutation in the cDNA sequence; CDS_position: relative position of the mutation in coding sequence; Protein_position: relative position of the amino acid in the protein. Amino_acids: only given if the variant affects the protein-coding sequence; Codons: the alternative codons with the variant base in upper case; QUALIFIER: Defines the relationship between Gene product and GO term; GO.TERM: Unique, stable identifier of the gene ontology term; GO.NAME: Gene ontology name; GENE_PRODUCT_NAME: Name of the gene product.

Supplementary Table S1. Description of the terms defined by the “Consequence“ column in Supplementary Data S1 tables.

Supplementary Table S2. Chromosome ("CHROM"), start position ("POS"), reference base(s) at the given position on the *S. aureus* strain Newman reference sequence ("REF"), mutated sequences ("ALT"), number of reads covering the mutations across samples ("DP") and fraction of the reads that contain the specific mutation across samples ("DP_Frac").
